# FFA-DMRI: A Network Based on Feature Fusion and Attention Mechanism for Brain MRI Denoising

**DOI:** 10.3389/fnins.2020.577937

**Published:** 2020-09-16

**Authors:** Dan Hong, Chenxi Huang, Chenhui Yang, Jianpeng Li, Yunhan Qian, Chunting Cai

**Affiliations:** ^1^School of Informatics, Xiamen University, Xiamen, China; ^2^Department of Neurology, The First Affiliated Hospital of Xiamen University, Xiamen, China

**Keywords:** magnetic resonance imaging, brain, denoising, feature fusion, attention mechanism

## Abstract

Magnetic Resonance Imaging (MRI) is an indispensable tool in the diagnosis of brain diseases due to painlessness and safety. Nevertheless, Rician noise is inevitably injected during the image acquisition process, which leads to poor observation and interferes with the treatment. Owing to the complexity of Rician noise, using the elimination method of Gaussian to remove it does not perform well. Therefore, the feature fusion and attention network (FFA-DMRI) is proposed to separate noise from observed MRI. Inspired by the attention-guided CNN network (ADNet) and Convolutional block attention module (CBAM), a spatial attention mechanism has been specially designed to obtain the area of interest in MRI. Furthermore, the feature fusion block concatenates local with global information, which makes full use of the multilevel structure and boosts the expressive ability of network. The comprehensive experiments on Alzheimer’s disease neuroimaging initiative dataset (ADNI) have demonstrated high effectiveness of FFA-DMRI with maintaining the crucial brain details. Moreover, in terms of visual inspections, the denoising results are also consistent with human perception.

## Introduction

Magnetic Resonance Imaging (MRI) of brains, with the superior features of non-radiation, non-invasiveness, and high resolution, is notable for diagnosis and treatment (Ikram et al., 2019; Jiang et al., 2019; Yu et al., 2019; Tripathi and Bag, 2020). In clinical practice, high-quality MRI can provide clear structural and functional information on brain tissues. However, noise is introduced into the raw image due to the circulation of magnetic fields and the interaction of magnets in MRI machines, which may hide the details of brain tissues and hinder the auto-computerized analysis (Jiang et al., 2017). Therefore, noise removal is a vital task to recover the clean MRI before the images are applied to diagnosis.

Previous research has established that the noise in MRI is governed by the Rician distribution, in which both real and imaginary parts are corrupted by Gaussian noise with equal variance (Bhadauria and Dewal, 2013; Li et al., 2020). The Rician distribution is signal-dependent as distinct from additive Gaussian noise. In other words, Rician noise is related to the image, and utilizing Gaussian denoising methods directly to remove it usually yields poor results. Thus, the right way to separate noise from the raw MRI without losing critical details is a huge challenge (Cai et al., 2020a).

With the increasing demand for image quality, a number of methods have been proposed for denoising. Existing methods can be mainly classified into two categories: transform domain methods and filtering methods (Mohan et al., 2014). The purpose of the transform domain method is to convert the original signal into a pattern that can remove noise more easily. For instance, a bilateral filtering scheme was proposed based on wavelets, in which the noise coefficient is expressed effectively by an undecimated wavelet transform (UDWT). There is a nice trade-off between the effect of noise removal and feature retention (Anand and Sahambi, 2010; Cai et al., 2020b). Based on wavelet shrinkage, the iterative scheme estimates the signal wavelet coefficients from the noisy images (Yu and Zhao, 2008). For signal high-dimensional singularities, wavelet transform does not perform well. Curvelet transform makes up for the shortcomings (Mohan et al., 2014). In this transform, edge directions are reproduced using the directivity and anisotropy of the curve (Do and Vetterli, 2005). However, the wavelet transform fails to resolve the curve with smooth edges. To overcome the drawback, a geometrical image transform was proposed, which greatly captures contours and details in MRI.

The filter methods, generally grouped into linear and non-linear parts, are adapted to remove noise in MRI. For linear filters, spatial filters and temporal filters are commonly employed (McVeigh et al., 1985; Mohan et al., 2014). Relatively, a spatial filter decreases the variance in MRI; however, it faces shortcomings in that it introduces the blurring of edges, which results in part of the required information that cannot be restored correctly (Soomro and Gao, 2016). Temporal filters are utilized only to spin-echo images. Furthermore, to prevent the aliasing artifacts, it is essential to select the appropriate filter to match filter sampling intervals. If the filters are too broad or too narrow, the performance is not satisfactory. For non-linear spatial filters, using a linear approach directly is not allowed. There are some typical examples in non-linear filters such as anisotropic diffusion filter (ADF) (Sijbers et al., 1999) and non-local means (NLM) (Coupé et al., 2006). The ADF approach obtains the denoising images efficiently with sharp edges. The filter of NLM employs redundant information to restore noise-free images. On the basis of the filter, unbiased NLM (Manjón et al., 2008) is exploited to improve the SNR in MRI; meanwhile, it does not influence the obvious structures. Nevertheless, the method has the shortcoming of high computational complexity.

Recently, methodologies based on deep learning are used to alleviate the above problem, such as deep plug-and-play super-resolution (DPSR) (Zhang K. et al., 2019), fast and flexible denoising convolutional neural network (FFDNet) (Zhang et al., 2018), and variance-stabilizing transformation inspired networks (VST-net) (Zhang M. et al., 2019). VST-net inherits the structures of traditional variance-stabilizing transformation and optimizes non-linear transformation through the design of a deep learning network. That shows the great potential of deep learning for denoising tasks. It is noted that the denoising convolutional neural network (DnCNN) (Zhang et al., 2017a) utilizes batch normalization and residual learning, which exhibits high effectiveness in JPEG image deblocking, single image super-resolution, and Gaussian denoising. Numerous deep learning methods for denoising have achieved outstanding performance. However, most research up to now has focused on the reduction of Gaussian noise, real noise, and blind noise. To our best knowledge, far too little attention has been paid to removing the Rician noise in MRI.

In this work, we propose a feature fusion and attention network (FFA-DMRI) for removing Rician noise in magnetic resonance (MR) images. Inspired by the structure of attention-guided CNN network (ADNet) (Tian et al., 2020), we have designed the FFA-DMRI network to restore noise-free images while maintaining critical brain tissues to the maximum extent possible. The main contributions of this paper are as follows:

(1) The proposed FFA-DMRI is dedicated to removing Rician noise in MR images. In contrast to other deep leaning methods for denoising, we specifically develop a spatial attention mechanism to focus on the area-of-interest of the brain.
(2) FFA-DMRI network is constructed with three blocks, including the feature extraction block, the feature fusion block, and the attention block. The feature extraction block utilizes the common convolution and the dilated convolution, which expands the receptive field and gains the details effectively. The feature fusion block is designed to combine the local and global features. Consequently, this block obtains more contextual information and promotes the reconstruction of pixels in MR images.
(3) The FFA-DMRI network is very superior for denoising on the ADNI dataset. In comparative experiments, it is competitive in quantitative metrics in terms of SSIM and PSNR. From the visual inspection, the denoising results are also in line with the human sense.

## Materials and Methods

### Rician Noise in MRI

The raw image generated by magnetic resonance equipment is *K*-space, including the real channel *P*_*r*_and the imaginary channel*P*_*i*_. Both channels are governed by Gaussian noise with equal variance σ^2^ and a mean value of zero (Zhu et al., 2009), which can be given by

(1) $$\left\{ \begin{array}{c} P_{\text{r}}=R\,\cos\alpha +ℜ\\ P_{i}=R\,\sin\alpha +ℑ \end{array}\right.$$

where *R* is the amplitude and α is the phase of raw signal. In addition, ℜ and ℑ denote the independent Gaussian noise which is injected into the real and the imaginary channel, respectively. An inverse discrete Fourier transform (Briggs and Henson, 1995) and the modular operation are exploited to reconstruct the MR images, which satisfies the human visual sense. The modular operation can be expressed as follows:

(2) $$D=\sqrt{P_{r}^{2}+P_{i}^{2}}=\sqrt{{\left(R\,\cos\alpha +ℜ\right)}^{2}+{\left(R\,\sin\alpha +ℑ\right)}^{2}}$$

After the non-linear transformation, the noise distribution is converted from Gaussian to Rician (He and Greenshields, 2008). The probability distribution function (PDF) of Rician noise can be estimated as

(3) $$p\,\left(D|R,\sigma \right)=\frac{D}{\sigma^{2}}\,e^{-\frac{D^{2}+R^{2}}{2\,\sigma^{2}}}\,I_{0}\,\left(\frac{R\,D}{\sigma^{2}}\right)$$

where *I*_*0*_ stands for the zeroth-order modified Bessel function (Sijbers and den Dekker, 2004) when the discrete grid is utilized to define MRI. From the PDF, it can be inferred that Rician noise is associated with images. For images with different signal-to-noise ratios (SNR), the distributions of Rician noise are disparate. If the value of SNR is relatively high, Rician distribution degenerates into a Gaussian distribution. Conversely, it tends to the Rayleigh distribution in low SNR. Therefore, compared with Gaussian noise, Rician noise is more complicated.

### Proposed Method

#### Network Architecture of FFA-DMRI

**FIGURE 1:**
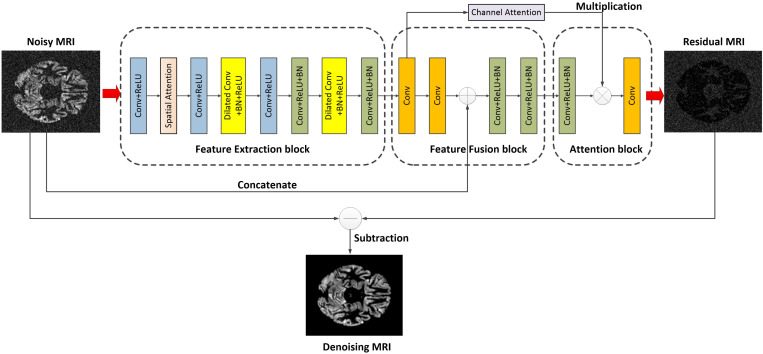
Overall architecture of the proposed FFA-DMRI.

Inspired by ADNet (Tian et al., 2020), the FFA-DMRI is proposed to eliminate noise in MRI. Figure 1 illustrates the overall architecture of FFA-DMRI. The network constructed by three sub-networks is as follows: a feature extraction block, a feature fusion block, and an attention block. These blocks correspond to the stages of denoising. Firstly, it employs common convolutions and dilated convolutions to expand the receptive field and acquires the features adequately. Furthermore, the operation of concatenation between global and local information enhances the expressive ability of network. Finally, the attention mechanism guides the network to extract useful information by assigning weights to different spatial positions and channels. The output of the network is the residual MR image and the potential clean image is obtained by subtracting the residual image from the input noisy image.

According to the structure of FFA-DMRI, the input of the network is noisy observed MRI, which is defined as*S*. The FFA-DMRI aims to learn the residual image *N* as an output rather than the potential clean image*C*. Every block is assumed to be a function; hence the execution process of network is defined as

(4) $$N=g_{a\,t}\,\left(g_{f\,u}\,\left(g_{e\,x}\,\left(S\right),S\right)\right)$$

where *g*_*ex*_, *g*_*fu*_, and *g*_*at*_ denote the functions of the feature extraction, feature fusion, and attention block, respectively. The output *N* is the mapping of noise in MRI and the potential clean image can be reconstructed by subtracting *N* from *S*. The implementations can be expressed as

(5) C=S-N

#### Feature Extraction Block

**FIGURE 2:**
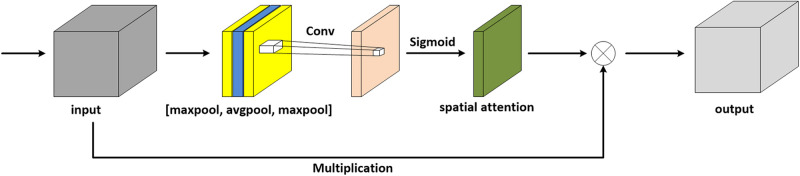
Spatial attention module with an additional layer of maximum pooling.

It is known that the crucial structural information in complex images is easily hidden, which leads to poor performance in practice. Therefore, extracting the representative features is notoriously hard but vital in deep learning. To overcome this problem, during the course of training, the network of FFA-DMRI is supposed to focus on the interest area of the brain and suppress the insignificant region. Motivated by that, we have designed the spatial attention mechanism inspired by CBAM (Woo et al., 2018), which is suitable for MR images specifically. Figure 2 depicts the spatial attention module with an additional layer of maximum pooling.

**FIGURE 3:**
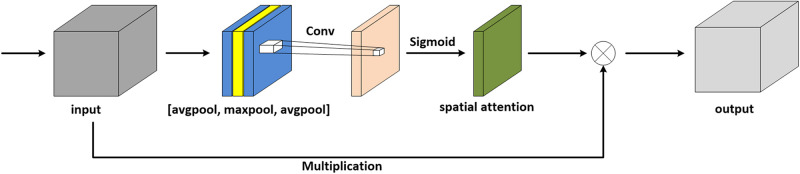
Spatial attention module with an additional layer of average pooling.

The attention module simulates the prioritization of visual information in human perception. In order to make the network pay more attention to the extraction of brain structure, an additional layer of maximum pooling is concatenated on the original architecture of CBAM. In this work, we consider another situation in which a spatial attention module is added with a layer of average pooling, as shown in Figure 3.

In terms of MR images, the pixels in the background are mostly black, and thus the values are zero. With respect to the brain regions, the pixel values are mostly greater than zero. For average pooling, the operation preserves background information and is suitable for the images where all pixels contribute to the prediction. Thus, average pooling is less applicable to MRI denoising. Relatively, maximum pooling is utilized to extract textures and assists the network in focusing on the brain regions, hence maximum pooling is selected in the module. To sum up, we take advantage of spatial attention to enhance the ability to extract brain features, which results in spending a lower amount of computing resources and achieving outstanding effects.

The batch normalization (BN) operation normalizes the input data; hence, it will destroy the original contrast of MR images. Besides, it has been pointed out that BN is more suitable to map data with regular distribution (Li et al., 2020). From the generation of Rician noise, it can be determined that the noise is non-linear. Thus, we do not employ the BN operation in the first two convolutions. Furthermore, the extraction of the context plays a crucial role in computer vision applications. For the denoising task, the construction of pixels is closely related to the context information (Yu and Koltun, 2016). In order to obtain more context, dilated convolution is utilized for the network, which enlarges the receptive field without reducing image resolution and losing details. Numerous works have been reported in the validity of dilated convolution (Yu and Koltun, 2016; Wei et al., 2018). For example, compared with a common convolution-based 3×3 kernel, a dilated convolution can serve a 5×5 or greater receptive field, but no increase in the number of parameters and computations. In FFA-DMRI, we integrate the common convolution and dilated convolution to take full advantage of information.

#### Feature Fusion Block

AlexNet (Krizhevsky et al., 2012), VGG-Net (Simonyan and Zisserman, 2014), and other deep learning models yield excellent results by increasing network layers. Nevertheless, on the one hand, the deeper network presents the phenomenon of gradient explosion and gradient disappearance. On the other hand, with continuous convolution, the effect of shallow features on a deep layer grows weak gradually. Thus, the way to extract high-quality features is pivotal for denoising tasks. To cope with the problem, we apply a lightweight and efficient feature-fusion module to combine low-level and high-level features. The module concatenates the intermediate feature map with the noisy observed MRI at the same scale, which makes full use of the structural information in the shallow network and boosts the network performance.

At the end of the feature fusion block, the two layers employ convolutions with the Rectified Linear Unit activation function (ReLU) and batch normalization. Compared with the sigmoid function, the ReLU function greatly reduces network computation and avoids the problem of gradient disappearance. Additionally, the ReLU function increases the non-linear relationship between the network layers, and thus it is appropriate to process the non-linear Rician noise. In this block, BN yields the distribution of images more stable, which greatly simplifies parameter adjustment and alleviates the problem of gradient disappearance.

#### Attention Block

**FIGURE 4:**
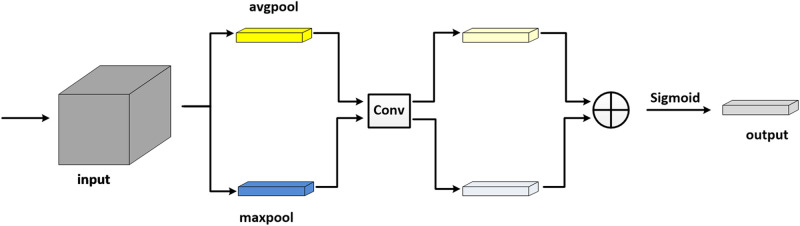
Channel attention module.

In computer vision, the attention mechanism improves the efficiency and accuracy of network to a certain extent. It adjusts the weight of each channel through training in order to enhance the influence of useful channels and suppress the unnecessary channels. Exploring the relationship between channels is beneficial to extract more vital content for the results and improve the denoising performance. In this paper, we exploit the maximum pooling and average pooling to the input feature map first. Furthermore, the two pooling layers are convolved separately. Then a sum of the convolutional layers yields the channel weights (Woo et al., 2018). The structure of channel attention is shown in Figure 4.

#### Loss Function

The loss function guides the further training of the network, and thus the selection of a loss function is directly related to the effect of execution. Different from the existing denoising networks that predict potential clean images directly, FFA-DMRI is able to estimate the residual images. Then subtracting the residual image from the input original image can obtain the clean images. Therefore, we use the mean square error (MSE) (Ephraim and Malah, 1984) to calculate the gap between the residual images generated by FFA-DMRI and the desired residual images. The desired residual image is obtained by subtracting noise-free image from noisy observed image. The loss function is described as

(6) $$L\,\left(\theta \right)=\frac{1}{2\,M}\,\sum_{k=1}^{M}{||f_{F\,F\,A-D\,M\,R\,I}\,\left(S_{k}\right)-\left(S_{k}-G_{k}\right)||}^{2}$$

Where *S* represents the noisy observed image and G stands for the noise-free image. θ denotes the parameter of FFA-DMRI training. *M* is the number of noisy-clean training image pairs.

## Experiments

### Data Acquisition and Training Settings

Deep learning is a data-driven technology. In other words, it requires a large amount of data for training to achieve promising performance. The network of FFA-DMRI is evaluated on the public real brain database of the Alzheimer’s disease neuroimaging initiative (ADNI)^1^. In our experiments, it consists of 199 three-dimensional (3D) images of brain MRI. We slice each 3D image to get the axial plane and select the slices that range from 37 to 86 due to less information in the head and tail regions. Then Rician noise is injected into images with noise levels of 5, 10, 20, and 30 according to formula (2), respectively. All the images have a resolution of 145×121, and they are divided into three parts; the training set contains 7,800 images, the test set includes 975 images, and the validation set consists of 975 images.

The network is trained with the PyTorch framework in Python and employs the NVIDIA GeForce GTX 960. In some scenarios, adaptive moment estimation (Adam) has better performance than the stochastic gradient descent (SGD) (Zhang, 2018). Thus, the optimizer used in this experiment is Adam (Kingma and Ba, 2014). The initial learning rate is chosen as 0.0001, and it is reduced by 0.5, 0.25, and 0.125 in the following training. The batch size is set to four due to the trade-off between GPU memory and computational speed.

### Qualitative Metrics

There are two popular qualitative metrics to evaluate denoising methods, including peak signal-to-noise ratio (PSNR) and structural similarity index measure (SSIM) (Kala and Deepa, 2018; Yu et al., 2019). PSNR calculates the distortion between recovered images *q*(*x*,*y*) and ground truth*p*(*x*,*y*). It can be defined as

(7) $$P\,S\,N\,R\,\left(p,q\right)=10\,\log_{10}\frac{255^{2}\times M\times N}{\sum_{\left(x,y\in \Omega \right)}{\left|p\,\left(x,y\right)-q\,\left(x,y\right)\right|}^{2}}\,\left(d\,B\right)$$

where M×*N* is the size of MR images and higher PSNR means the less distortion in images.

The metrics of SSIM is based on three comparative measurements, including luminance, contrast, and structure. Itis more consistent with human visual perception (Wang et al., 2004), that can be obtained by

(8) $$S\,S\,I\,M\,\left(p,q\right)=\frac{\left(2\,u_{p}\,u_{q}+c_{1}\right)\,\left(2\,\sigma_{p\,q}+c_{2}\right)}{\left(u_{p}^{2}+u_{q}^{2}\right)\,\left(\sigma_{p}^{2}+\sigma_{q}^{2}+c_{2}\right)}$$

where *u*_*p*_, *u*_*q*_ is the average of *p*, *q*, respectively. And $\sigma_{p}^{2}$ is the variance of *p*; $\sigma_{p}^{2}$ denotes the variance of *q*, and σ_*pq*_ represents the covariance of *p* and *q*. To avoid instability, SSIM appends two constants including *c*_*1*_ and *c*_*2*_. The value range of SSIM is [0,1].

### Performance Comparison

In order to verify the effectiveness of the proposed FFA-DMRI, comparative experiments are conducted under the same dataset and parameters to guarantee fairness. The results of different denoising schemes are evaluated in terms of quantitative and qualitative metrics. Collectively, we compare the proposed network FFA-DMRI with common denoising algorithms, including NLM (Buades et al., 2011), BM3D (Danielyan et al., 2011), MRF (Ji, 2019), Wiener filter (Jang and Kim, 2001), WNNM (Gu et al., 2014), IRCNN (Zhang et al., 2017b) and DnCNN (Zhang et al., 2017a).

#### Quantitative Metrics

Based on the above Settings, the average PNSR results of different denoising methods are presented in Table 1, and the average SSIM results are reported in Table 2.

**TABLE 1:** The average PSNR/dB results of different methods on the ADNI dataset at different noise levels.

Noise level λ	BM3D	NLM	Wiener filter	MRF	WNNM	IRCNN	DnCNN	FFA-DMRI (ours)
5	28.57	31.90	21.59	25.92	33.00	39.09	39.72	**39.76**
10	25.52	26.68	20.79	23.89	27.08	34.31	34.83	**35.23**
20	21.05	21.13	18.81	20.28	21.28	29.12	30.24	**30.55**
30	18.27	18.01	17.03	18.27	17.97	26.40	27.32	**27.51**

*The best results at each noise level are highlighted in bold.*

**TABLE 2:** The average SSIM results of different methods on the ADNI dataset at different noise levels.

Noise level λ	BM3D	NLM	Wiener filter	MRF	WNNM	IRCNN	DnCNN	FFA-DMRI (ours)
5	0.5261	0.5477	0.3314	0.4897	0.5633	0.9914	0.9935	**0.9946**
10	0.4439	0.3850	0.2637	0.4117	0.4768	0.9797	0.9815	**0.9850**
20	0.3701	0.4654	0.2125	0.3423	0.3839	0.9320	0.9507	**0.9586**
30	0.3084	0.3143	0.1762	0.3084	0.2700	0.8907	0.9064	**0.9166**

*The best results at each noise level are highlighted in bold.*

It is known that the Rician noise depends on the images, so removing it is more complicated than Gaussian additive noise. From Table 1, the best result of PSNR at each noise level is shown in bold. It can be observed that the proposed FFA-DMRI outperforms other methods tested. For BM3D and NLM methods, it is difficult to match similar regions at higher noise levels. Besides, searching and matching regions consume much time. Therefore, the traditional method does not perform well on the dataset. It is noted that deep learning methods achieve outstanding denoising results. In particular, among the listed deep learning methods, the proposed FFA-DMRI promotes the removing performance at each noise level. On the specifics, FFA-DMRI exceeds IRCNN 1.43 dB at the noise level of 20 and is superior to DnCNN at every noise level. Additionally, we measured the results of the above methods in terms of SSIM.

The metrics of SSIM indicates that the structural similarity between recovered images and ground truth. As described in Table 2, FFA-DMRI achieves the best performance. It brings an improvement of 2.66% than IRCNN at the noise level of 20. Note that when the noise level is 5, our method tends to the highest value (the highest value in SSIM is 1), which shows that recovered images perfectly restore noise-free images. Besides, the value of SSIM is over 90% at each noise level for FFA-DMRI. Consistent with the results of PSNR, the traditional methods are inferior to the deep learning methods in this dataset. In summary, there is a significant improvement in brain MRI denoising, which reconstructs the latent clean image with maintaining the vital structure information. Thus FFA-DMRI is a competitive denoising method in terms of quantitative analysis.

#### Qualitative Metrics

**FIGURE 5:**
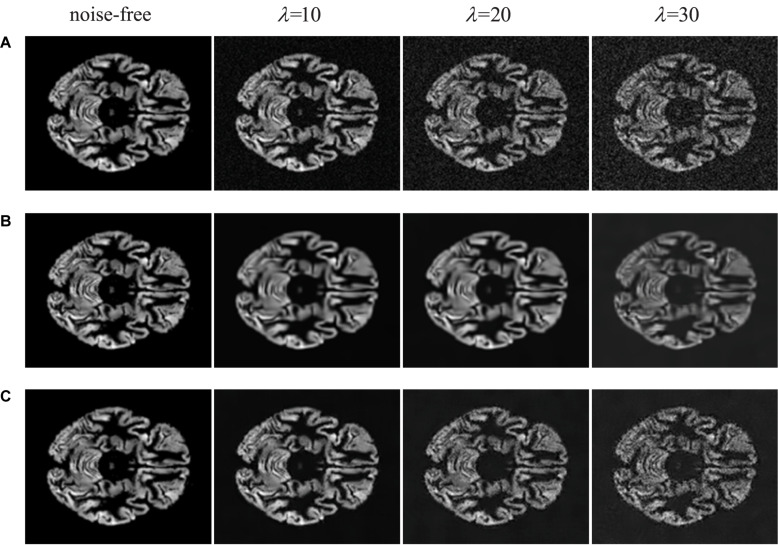
Visual inspections of brain MRI denoised by traditional methods. **(A)** MRI with noise of different levels. **(B)** The results of BM3D at different noise levels. **(C)** The results of NLM at different noise levels.

**FIGURE 6:**
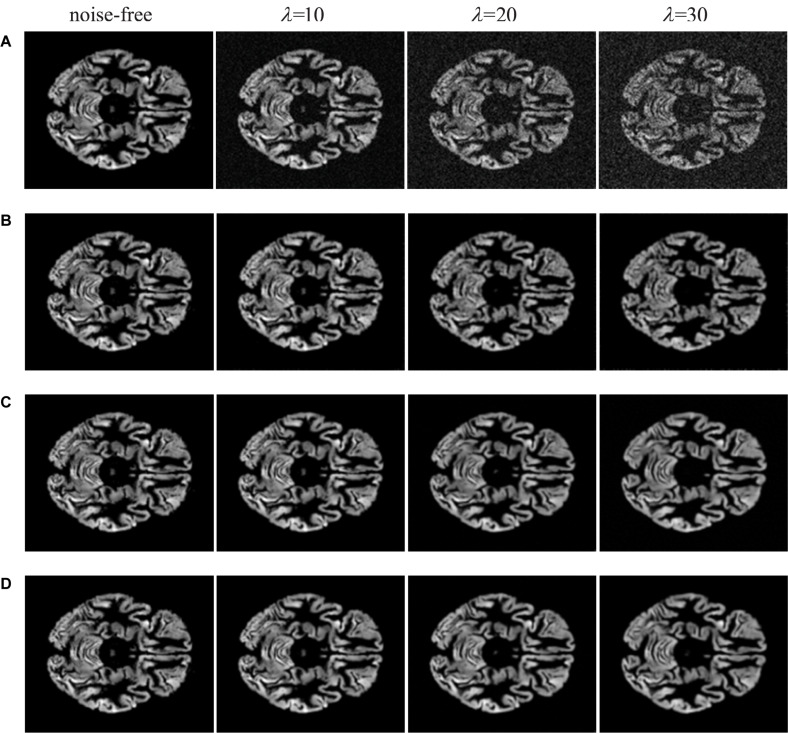
Visual inspections of brain MRI denoised by deep learning methods. **(A)** MRI with noise of different levels. **(B)** The results of IRCNN at different noise levels. **(C)** The results of DnCNN at different noise levels. **(D)** the results of FFA-DMRI at different noise levels.

In practice, it is indispensable to evaluate image quality through the human senses. In some cases, the metrics of SSIM and PSNR are outstanding in computer vision tasks; however, the images that do not satisfy human perception are distorted. In this paper, we list the visual inspections of comparative experiments as illustrated in Figures 5, 6.

From Figure 5, we illustrate traditional denoising methods for comparison. It is noticeable that the BM3D and NLM methods can remove a part of noise while generating the blurred structure of brain. Meanwhile, some crucial details in original images are lost. When the concentration of noise increases, the removal effect is worse on speediness with poor visual perception. In Figure 6, visual illustrations of deep learning methods are shown. It can be seen that the effect of deep learning methods is superior to traditional methods, which remove background noise and recover most of the complex brain structures.

In comparison with IRCNN, the proposed FFA-DMRI yields clearer brain tissues and sharper edges after noise removal. The method of DnCNN also achieves excellent results in the experiments; however, FFA-DMRI maintains more subtle features of the original image, and the contrast between brain regions and background is stronger than DnCNN. The obvious contrast contributes to enhancing the interpretation and recognition of images and satisfies the needs of clinical analysis. In general, FFA-DMRI we proposed performs well in the quantitative and qualitative analysis.

## Conclusion

In this article, we propose a network to remove Rician noise from a brain MRI as well as FFA-DMRI. The network is composed of a feature extraction block, a feature fusion block, and an attention block. The feature extraction block exploits the spatial attention mechanism to obtain the area of interest emphatically. Moreover, we utilize dilated convolutions, which expand the receptive fields, and we fuse local and global information to boost the network performance. Then the channel attention mechanism is employed to enhance the influence of essential elements and suppress the useless channels. After the above steps are carried out, FFA-DMRI is trained on the ADNI dataset. In terms of quantitative evaluation, SSIM and PSNR are adopted. Experimental results show that FFA-DMRI can effectively remove Rician noise and maintain most of the crucial details. For quantitative evaluation, it can be seen from visual inspection that the recovered images are more consistent with human senses with obvious contrast, clear brain tissues, and sharp edges. Therefore, the proposed method FFA-DMRI is competitive in brain MRI denoising, which can assist clinicians in diagnosis and treatment.

## Data Availability Statement

Publicly available datasets were analyzed in this study. This data can be found here: http://adni.loni.usc.edu/.

## Author Contributions

DH was responsible for the work of writing the manuscript and doing experiments. CH, CY, JL, and YQ made the experiments. CC modified the English grammar of the article. All authors contributed to the article and approved the submitted version.

## Conflict of Interest

The authors declare that the research was conducted in the absence of any commercial or financial relationships that could be construed as a potential conflict of interest.

## Footnotes

1 http://adni.loni.usc.edu/
